# Working conditions, career barriers and perceived equity among neuro-oncology professionals: insights from the Japan society for neuro-oncology (JSNO) gender equality and diversity committee survey

**DOI:** 10.1007/s10147-026-03168-y

**Published:** 2026-08-18

**Authors:** Akiko Higuchi, Kaoru Tamura, Yuko Watanabe, Tatsuya Abe, Mitsutoshi Nakada, Shota Tanaka, Yoshiko Okita, Motoo Nagane, Yuichi Hirose, Takamitsu Fujimaki

**Affiliations:** 1 Department of Breast and Medical Oncology, National Center for Global Health and Medicine, Tokyo, Japan; 2 Department of Neurosurgery, Institute of Science Tokyo, 1-5-45 Yushima, Bunkyo-Ku, Tokyo, 113-8519 Japan; 3 Department of Pediatric Oncology, National Cancer Center Hospital, Tokyo, Japan; 4 Department of Neurosurgery, Faculty of Medicine, Saga University, Saga, Japan; 5 Department of Neurosurgery, Graduate School of Medical Science, Kanazawa University, Ishikawa, Japan; 6 Department of Neurological Surgery, Dentistry, and Pharmaceutical Sciences, Okayama University Graduate School of Medicine, Okayama, Japan; 7 Department of Neurosurgery, Faculty of Medicine, University of Miyazaki, Miyazaki, Japan; 8 Department of Neurosurgery, Kyorin University School of Medicine, Tokyo, Japan; 9 Department of Neurosurgery, Fujita Health University School of Medicine, Aichi, Japan; 10 Department of Neurosurgery, Saitama Medical University Hospital, Saitama, Japan

**Keywords:** Neuro-oncology, Work-life balance, Career development, Gender equity, Survey

## Abstract

**Background:**

Gender equity, work-life balance, and career development have become important concerns in the medical profession. However, empirical evidence on how neuro-oncology professionals experience working conditions, career barriers, and perceived inequity remains limited.

**Methods:**

We conducted a nationwide, cross-sectional, anonymous online survey of members of the Japan Society for Neuro-Oncology (JSNO) to evaluate working conditions, career-related concerns, job satisfaction, perceived workplace discrimination, and organizational challenges.

**Results:**

Among 292 respondents (response rate, 35.9%), the mean weekly working time was 56.7 h, and 45.5% worked ≥ 60 h per week. Women were markedly underrepresented in managerial/leadership positions (6.7% vs. 47.3%) and were more likely to hold part-time positions. Multivariable logistic regression analysis showed that female sex was independently associated with greater perceived workplace discrimination (OR 2.71, 95% CI [1.23–5.98], *p* = 0.013), after adjustment for working hours, caregiving experience, and managerial/leadership position. An age-by sex interaction was observed for career-related concerns: these increased with age among women but decreased among men. Longer working hours were associated with lower satisfaction with income, working hours, and work-life balance. Although workplace support systems are widely available, substantial perceptual gaps and workload redistribution challenges were identified.

**Conclusion:**

These findings highlight persistent gender disparities and structural workforce challenges in Japanese neuro-oncology, underscoring the need for institutional strategies to promote sustainable workforce development and gender equity.

**Supplementary Information:**

The online version contains supplementary material available at https://doi.org/10.1007/s10147-026-03168-y.

## Introduction

Gender equity, work-life balance, and career development are increasingly recognized as global priorities in medicine. In Japan, disparities in leadership representation, academic advancement, and work-life balance have been reported across multiple medical specialties [1–3]. These challenges are particularly relevant in neuro-oncology, a highly specialized field that depends on multidisciplinary collaboration among physicians, nurses, rehabilitation therapists, pharmacists, medical social workers, and researchers. The Gender Equality and Diversity Committee of the Japan Society for Neuro-Oncology (JSNO), known as Women and Diversity in Neuro-Oncology (WING), was established in 2020 to promote greater opportunities for diverse members, including women. In 2021, the committee conducted a survey of all members to assess working conditions, workplace climate, and career-related challenges among neuro-oncology professionals.

Although the increased participation of women in neuro-oncology has drawn attention to gender-related issues, it remains unclear whether perceived inequity is primarily attributable to gender or to structural differences in working conditions and professional roles. This study used quantitative and qualitative survey data to examine factors associated with perceived inequity, career-related concerns, and job satisfaction among neuro-oncology professionals in Japan.

## Materials and methods

### Study design and participants

This cross-sectional study was conducted using an anonymous web-based questionnaire distributed to all registered JSNO members between September 22 and October 12, 2021. The society includes physicians and allied healthcare professionals involved in neuro-oncology practice across Japan. Participation was voluntary, and electronic informed consent was obtained before participants initiated the survey. The study protocol was approved by the Ethics and Academic Committees of JSNO (JSNO2021-1).

### Survey instrument

The questionnaire collected data on demographic characteristics, employment status, weekly working hours, managerial status, caregiving experience (childbirth, childcare, and eldercare), and institutional characteristics. We assessed self-reported sex (male or female), rather than gender identity. Weekly working hours were self-reported; Values exceeding 120 h per week were treated as missing. Long working hours were defined as ≥ 60 h per week. Managerial/leadership positions were defined based on current appointments as a professor, associate professor, department head, or hospital leadership positions. Job satisfaction was assessed across six domains: income, position/status, working hours, use of skills and abilities, work-life balance, and relationships with supervisors and colleagues. Each domain was rated on a 5-point Likert scale (very satisfied, satisfied, neutral, dissatisfied, and very dissatisfied). Respondents selecting “very satisfied” or “satisfied” were classified as satisfied. Participants rated perceived discrimination using a five-point scale ranging from “very strongly” to “not at all.” For regression analyses, responses were dichotomized as “very strongly” or “often” versus all other responses, based on the response distribution and sample size. Additional items assessed career-related concerns; satisfaction with income, working hours, and work-life balance; availability of support systems; and perceived workload redistribution when colleagues took leave. Details of all questionnaire items are provided in the Supplementary Appendix (Questionnaire for the JSNO Gender Equality and Diversity Committee Survey).

### Statistical analysis

Descriptive statistics were calculated for all variables. Group differences were evaluated using chi-square or Fisher’s exact tests, depending on expected cell counts for categorical variables, and t-tests for continuous variables, as appropriate. Multivariate binary logistic regression was used to estimate adjusted odds ratios (ORs) and 95% confidence intervals (CIs) for perceived workplace discrimination and promotion-related inequities. Multivariate linear regression was used to examine the factors associated with overall job satisfaction. A two-sided *p*-value < 0.05 was considered statistically significant. Statistical analyses were performed using IBM SPSS Statistics (IBM Corp., Armonk, NY, USA) and JMP (SAS Institute Inc., Cary, NC, USA).

## Results

### Participant characteristics and working conditions

Among 814 society members, 292 (35.9%) responded. The demographic characteristics of the respondents are summarized in Table 1. Most respondents were male (262 men and 30 women). The response rate was 35.0% for men and 45.5% for women. The mean age was 48.6 years (range, 27–74 years), and most respondents (94.8%) were employed full-time. After excluding one unemployed female respondent, women were significantly more likely to work in part-time or non-full-time positions compared with men (16.7% vs. 3.8%; OR, 5.25; 95% CI, 1.64–16.83; *p* = 0.004; Fisher’s exact test). Physicians accounted for the majority of respondents (n = 278, 95.2%), followed by nurses (n = 4, 1.4%), and other professions (n = 10, 2.7%), including pharmacists, psychologists, social workers, rehabilitation professionals, radiologic technologists, and non-physician researchers. Of the 278 physicians, 250 were neurosurgeons, nine were radiologists, eight were pathologists, and six were pediatricians. Mean weekly working hours were 56.7 h ± 15.3 h, and 45.5% of respondents reported working ≥ 60 h per week. The mean number of night duties per month was 2.4 for men and 1.0 for women. Most respondents (85.3%) had children. Overall, 43.2% of respondents held managerial/leadership positions. Women were significantly less likely than men to hold managerial/leadership positions (6.7% vs. 47.3%; OR, 0.08; 95% CI: 0.02–0.35; *p* < 0.001; Fisher’s exact test).

**Table 1:** Participant characteristics and working conditions among neuro-oncology professionals

Characteristic	All (n = 292)	Men (n = 262)	Women (n = 30)
Age, years	48.6 (9.3)	49.1 (9.2)	43.6 (8.6)
Age group (years)			
20 s (20–29)	1 (0.3%)	1 (0.4%)	0 (0.0%)
30 s (30–39)	51 (17.5%)	39 (14.9%)	12 (40.0%)
40 s (40–49)	109 (37.3%)	95 (36.3%)	14 (46.7%)
50 s (50–59)	86 (29.5%)	84 (32.1%)	2 (6.7%)
60 s (60–69)	41 (14.0%)	39 (14.9%)	2 (6.7%)
70 s (70–79)	4 (1.4%)	4 (1.5%)	0 (0.0%)
Profession: Physician	278 (95.2%)	255 (97.3%)	23 (76.7%)
Profession: Nurse	4 (1.4%)	1 (0.4%)	3 (10.0%)
Profession: Pharmacist	1 (0.3%)	1 (0.4%)	0 (0.0%)
Profession: Other	9 (3.1%)	5 (1.9%)	4 (13.3%)
Work style: Full-time (regular employee)	276 (94.5%)	252 (96.2%)	24 (80.0%)
Work style: Part-time/non-full-time (contract/temporary/per diem/other)	15 (5.1%)	10 (3.8%)	5 (16.7%)
Weekly working hours	56.7 (15.3)	57.4 (15.0)	50.8 (16.9)
Working ≥ 60 h/week	133 (45.5%)	126 (48.1%)	7 (23.3%)
Night duties per month	2.2 (2.6)	2.4 (2.6)	1.0 (1.4)
Has children	249 (85.3%)	235 (89.7%)	14 (46.7%)
Has caregiving experience	39 (13.4%)	33 (12.6%)	6 (20.0%)
Supportive workplace (Yes)	146 (50.0%)	130 (49.6%)	16 (53.3%)
Managerial/leadership position	126 (43.2%)	124 (47.3%)	2 (6.7%)

Values are presented as mean (standard deviation), median (interquartile range), or number (%), as appropriate

### Concerns regarding career development and perceived barriers

Career-related concerns were reported by 33.2% of male respondents and 56.7% of female respondents (Table 2A). A significant association was observed between sex and career-related concerns (χ^2^(2, *N* = 292) = 7.53; *p* = 0.023), with women more likely than men to report such concerns, although the effect size was small (R^2^ = 0.012). “Physical condition or stamina” and “personal abilities or skills” were frequently reported as barriers to career development by both male and female respondents. Women more frequently reported “workplace interpersonal relationships,” “childbirth and childcare,” “workplace gender discrimination,” and “spouse’s job transfer or overseas assignment” as barriers to career development (Table 2B). Multivariable logistic regression analysis, including an age-by-sex interaction term, demonstrated a significant interaction (Table 2C). Career-related concerns decreased with age among men but increased with age among women (Supplementary Fig. 1).

**Table 2:** Concerns regarding career development and perceived barriers

(A) Do you have concerns regarding your career development?			
Response	All (n = 292) (%)	Men (n = 262) (%)	Women (n = 30) (%)
Yes	35.6	33.2	56.7
No	40.1	42.4	20.0
Not sure	24.3	24.4	23.3

(B) Factors that hinder career development					
Barrier	Total (n = 292) (%)	Men (n = 262) (%)	Women (n = 30) (%)	*p* value	Test
Physical condition or stamina	36.0	35.9	36.7	1	χ^2^
Personal abilities or skills	46.6	46.2	50.0	0.81	χ^2^
Poor working conditions	24.7	25.6	16.7	0.408	χ^2^
Lack of access to professional education at the workplace	6.5	6.5	6.7	1	Fisher
**Workplace interpersonal relationships**	19.9	17.9	**36.7**	**0.027**	χ^2^
Difficulty finding suitable employment	14.0	14.1	13.3	1	Fisher
Marriage	2.7	2.7	3.3	0.582	Fisher
**Childbirth and childcare**	7.5	5.7	**23.3**	**0.003**	Fisher
Children’s education	1.0	0.4	6.7	0.438	χ^2^
**Workplace gender discrimination**	16.8	16.0	**23.3**	**0.028**	Fisher
Lack of family support	3.1	2.7	6.7	0.231	Fisher
**Spouse’s job transfer or overseas assignment**	4.1	3.1	**13.3**	**0.025**	Fisher
Financial issues	18.5	19.5	10.0	0.317	χ^2^
Other	8.9	11.8	13.3	1	Fisher

(C) Multivariable logistic regression analysis of factors associated with concerns regarding career development			
Variable	Odds ratio (OR)	95% CI	*p* value
Age (per 1-year increase)	0.92	0.89–0.95	< 0.001
Sex (female vs male)	0.0004	0.00002–0.12	0.007
Age × sex interaction	1.21	1.06–1.38	0.004

Values are percentages. Multiple responses were allowed

Because of the marked imbalance in sample size between groups, Fisher’s exact test was applied when expected cell counts were small to ensure accurate estimation of *p* values

Concerns regarding career development were coded as yes = 1 and no = 0. Age was treated as a continuous variable. Sex was coded as female = 1 and male = 0. Odds ratios (ORs) and 95% confidence intervals (CIs) were estimated using multivariable logistic regression

### Job satisfaction among Neuro-oncology professionals

**Fig. 1:**
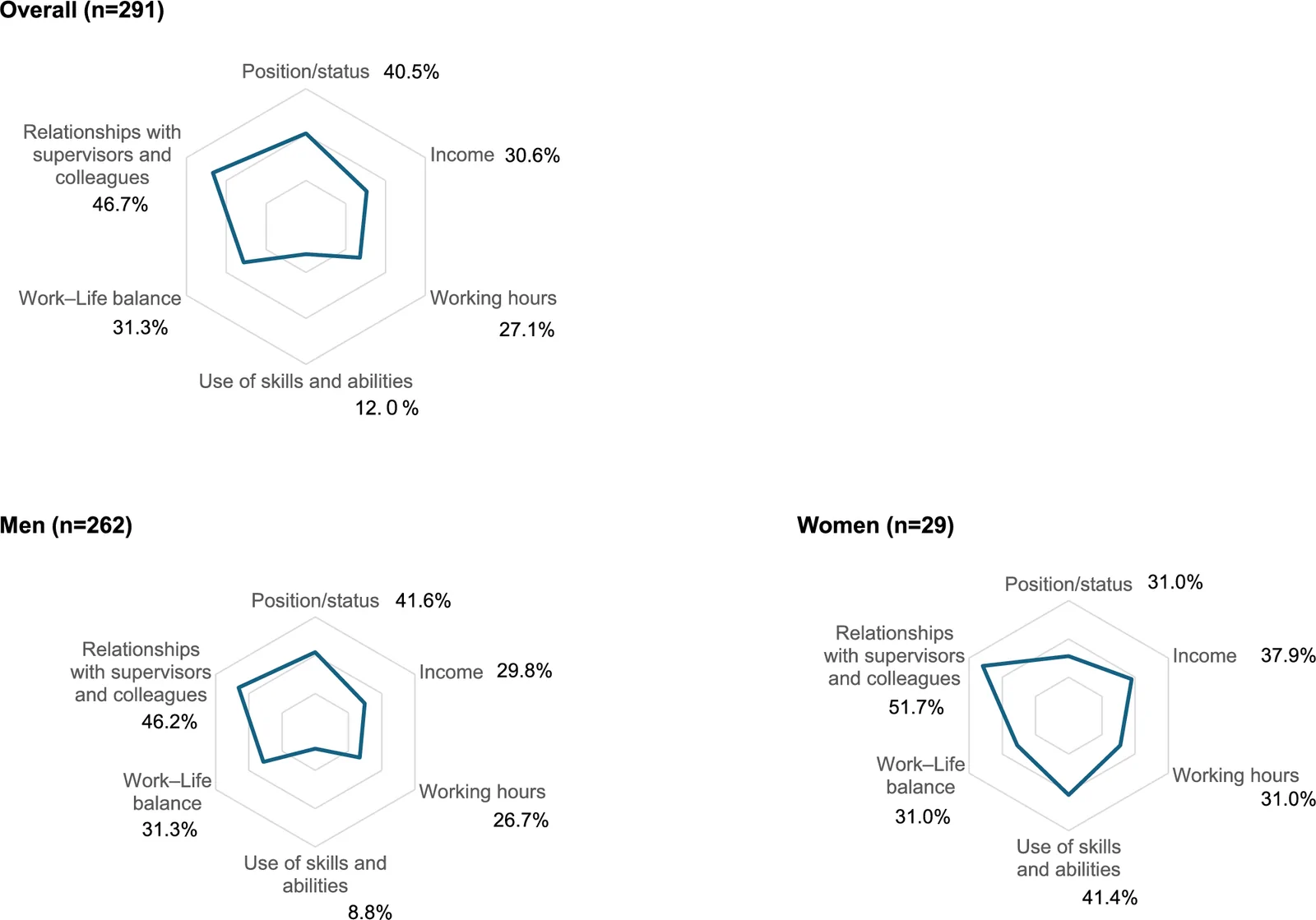
Job satisfaction across six domains, overall and stratified by sex. Radar charts illustrate the proportion of respondents who answered “very satisfied” or “satisfied” across six domains: position/status, income, working hours, use of skills and abilities, work–life balance, and relationships with supervisors and colleagues. The results are shown for the overall sample (n = 291, excluding missing responses) and separately for men (n = 262) and women (n = 29). Percentages represent the proportions within each subgroup. Missing responses are excluded from the denominator

Figure 1 illustrates job satisfaction across six domains, overall and by sex. Among all respondents, 40.5% were satisfied with their job position/status, 30.6% with income, 27.1% with working hours, 12.0% with their use of skills and abilities, 46.7% with relationships with superiors or colleagues, and 31.3% with work-life balance. No significant sex differences were observed in satisfaction with income (29.8% vs. 37.9%; *p* = 0.40), working hours (26.7% vs. 31.0%; *p* = 0.66), work-life balance (31.3% vs. 31.0%; *p* = 1.00), or professional relationships (46.2% vs. 51.7%; *p* = 0.70). In contrast, female respondents were significantly more likely than male respondents to report satisfaction with use of skills and abilities (41.4% vs. 8.8%; *p* < 0.001). In multivariable logistic regression analysis, long working hours were significantly associated with lower satisfaction with income, working hours, and work-life balance (Table 3). Conversely, holding a managerial/leadership position was significantly associated with greater satisfaction with income, working hours, and job position (Table 3).

**Table 3:** Factors associated with job satisfaction across six domains

(A) Satisfaction with income			
Predictor	OR	95% CI	*p* value
Sex (female vs male)	1.44	0.60–3.43	0.415
Caregiving experience (yes vs no)	1.71	0.83–3.52	0.145
Long working hours (≥ 60 h/week vs < 60 h/week)	**0.43**	**0.24–0.75**	**0.003**
Managerial/leadership position(yes vs no)	**1.77**	**1.02–3.06**	**0.042**

(B) Satisfaction with position/status			
Predictor	OR	95% CI	*p* value
Sex (female vs male)	1.2	0.51–2.86	0.677
Caregiving experience(yes vs no)	1.14	0.56–2.34	0.719
Long working hours(≥ 60 h/week vs < 60 h/week)	0.6	0.36–1.01	0.055
Managerial/leadership position(yes vs no)	**3.29**	**1.96–5.54**	**< 0.001**

(C) Satisfaction with working hours			
Predictor	OR	95% CI	*p* value
Sex (female vs male)	1.25	0.50–3.08	0.635
Caregiving experience(yes vs no)	0.89	0.40–1.98	0.776
Long working hours(≥ 60 h/week vs < 60 h/week)	**0.29**	**0.15–0.54**	**< 0.001**
Managerial/leadership position(yes vs no)	**1.91**	**1.07–3.39**	**0.029**

(D) Satisfaction with use of skills and abilities			
Predictor	OR	95% CI	*p* value
Sex (female vs male)	0.84	0.37–1.92	0.683
Caregiving experience(yes vs no)	1.54	0.77–3.06	0.222
Long working hours(≥ 60 h/week vs < 60 h/week)	0.97	0.60–1.57	0.9
Managerial/leadership position(yes vs no)	1.3	0.80–2.12	0.283

(E) Satisfaction with work-life balance			
Predictor	OR	95% CI	*p* value
Sex (female vs male)	0.77	0.32–1.88	0.568
Caregiving experience(yes vs no)	1.85	0.90–3.78	0.094
Long working hours(≥ 60 h/week vs < 60 h/week)	**0.37**	**0.21–0.65**	**< 0.001**
Managerial/leadership position(yes vs no)	1.11	0.65–1.90	0.694

(F) Satisfaction with relationships with supervisors and colleagues			
Predictor	OR	95% CI	*p* value
Sex (female vs male)	1.22	0.55–2.74	0.627
Caregiving experience(yes vs no)	1.05	0.53–2.08	0.894
Long working hours (≥ 60 h/week vs < 60 h/week)	0.76	0.47–1.23	0.266
Managerial/leadership position(yes vs no)	1.14	0.70–1.84	0.607

OR, odds ratio; CI, confidence interval

Outcome was defined as satisfaction (“satisfied” or “very satisfied”) versus others (neutral, dissatisfied, or very dissatisfied). Multivariable logistic regression analyses were performed using complete-case data among respondents who provided consent. Odds ratios were adjusted for sex, caregiving experience, working hours (≥ 60 h/week), and managerial/leadership position.

### Factors associated with perceived workplace discrimination and promotion-related inequity

Among all respondents, 15.4% reported experiencing workplace discrimination, and 15.8% perceived promotion-related inequity. Female sex was independently associated with stronger perceived workplace discrimination (adjusted OR, 2.71; 95% CI: 1.23–5.98; *p* = 0.013, Table 4A). Caregiving responsibilities, long working hours, and managerial status were not significantly associated with perceived discrimination. For perceived promotion-related inequity, female sex was not significantly associated with the outcome (OR, 1.86; 95% CI: 0.87–3.96; *p* = 0.108, Table 4B). Managerial/leadership position showed a marginal inverse association (OR, 0.65; 95% CI: 0.42–1.01; *p* = 0.053, Table 4B). Caregiving experience and long working hours were not significantly associated with promotion-related inequity.

**Table 4:** Factors associated with perceived workplace discrimination and promotion-related inequity

(A) Perceived workplace discrimination			
Predictor	OR	95% CI	*p* value
Sex (female vs male)	2.71	1.23–5.98	**0.013**
Caregiving experience (yes vs no)	1.15	0.62–2.12	0.66
Long working hours (≥ 60 h/week vs < 60 h/week)	1.43	0.93–2.18	0.101
Managerial/leadership position (yes vs no)	0.88	0.57–1.36	0.576

(B) Perceived promotion-related inequity			
Predictor	OR	95% CI	*p* value
Sex (female vs male)	1.86	0.87–3.96	0.108
Caregiving experience (yes vs no)	0.9	0.49–1.66	0.747
Long working hours (≥ 60 h/week vs < 60 h/week)	1	0.65–1.53	0.996
Managerial/leadership position (yes vs no)	0.65	0.42–1.01	0.053

OR, odds ratio; CI, confidence interval

### Workplace support systems for childbirth, childcare and caregiving responsibilities and related emotional responses

Regarding workplace support systems for childbirth, childcare, and caregiving responsibilities, 80.1% of respondents reported access to maternity leave, 63.7% to childcare leave, and 34.9% to caregiving leave (Supplementary Table 1A). The perceived availability of workplace support systems was significantly higher among respondents in managerial/leadership positions than among those in non-managerial/leadership positions across multiple items, including maternity leave, exemption from night duty during pregnancy, childcare leave, employment protection during childcare leave, and exemption from radiation-related duties (Supplementary Table 1B). Overall, 43.8% of respondents reported experiencing an increased workload when colleagues took maternity, childcare, or caregiving leave, or used reduced working-hour arrangements (Supplementary Table 2A). Among those reporting increased workload, 63.6% expressed willingness to cooperate, whereas 16.4% perceived the situation was unfair (Supplementary Table 2B). Respondents working long hours (≥ 60 h per week) were significantly more likely both to express a willingness to cooperate and to perceive the situation as unfair (Supplementary Table 2C and 2D). Perceptions of workplace supportiveness differed significantly by managerial/leadership position (*p* = 0.036; Supplementary Table 3). A higher proportion of respondents in managerial/leadership positions reported that their workplace was supportive (53.7%) compared with those in non-managerial/leadership positions (46.2%). Regarding potential solutions to situations in which remaining staff experienced excessive workloads when colleagues use caregiving or childcare leave or related support systems, managerial respondents tended to emphasize staffing and system-level challenges, whereas non-managerial respondents highlighted personal workload, emotional burden, and the need for transparency and recognition (Supplementary Table 2E).

### Positive actions to improve work-life balance

Policies, systems, and support measures considered necessary to achieve a balance between work and childcare or caregiving responsibilities are summarized in Supplementary Table 4. Many respondents indicated that increasing the number of physicians, expanding the non-physician workforce, reviewing the primary physician system, and providing exemptions from night or on-call duties were necessary. Female respondents were significantly more likely than male respondents to report the need for exemption from overtime work, expansion of reduced working hours, provision of sick childcare services, expansion of after-school childcare programs, and promotion of men’s participation in housework, childcare, and caregiving.

## Discussion

This study provides the first comprehensive analysis of gender equity, work-life balance, career concerns, job satisfaction, and operational challenges among neuro-oncology professionals in Japan. The mean weekly working time of 56.7 h, with nearly half of respondents working ≥ 60 h per week, highlights the persistently heavy workload across this field. Most respondents to this survey were male and worked as neurosurgeons, consistent with the current composition of the neuro-oncology workforce in Japan. This pattern reflects the broader imbalance in sex in neurosurgery and related specialties that form the core of the neuro-oncology workforce. In Japan, neurosurgeons typically complete six years of postgraduate clinical training, including residency, before obtaining board certification and pursuing subspecialty training in areas such as neuro-oncology. Promotion to managerial/leadership positions generally occurs after many years of clinical and research experience. Consequently, managerial/leadership positions in our cohort were predominantly occupied by older male respondents. This career structure may also partially explain the advanced age of the study population. Notably, female respondents were substantially younger than male respondents, likely reflecting the increasing number of women entering neurosurgery in recent years. This age imbalance may have influenced perceptions regarding career development and promotion opportunities. Women were underrepresented in managerial/leadership positions and were more likely to hold non-full-time employment. These findings are consistent with prior reports from Japan describing structural barriers faced by women physicians [4, 5] as well as with international evidence demonstrating the persistent underrepresentation of women in academic leadership, even after adjustment for academic productivity and career duration [6]. The recent EANO Positive Action Initiative likewise highlighted gender imbalances and emphasized the need for structured institutional strategies within neuro-oncology [7]. Taken together, these findings suggest that the disparities observed in this study are not unique to Japan but instead reflect a broader global challenge in neuro-oncology.

In this study, female respondents reported more frequent concerns regarding career development than male respondents. A significant age-by-sex interaction was observed (OR, 1.21; 95% CI 1.06–1.38; *p* = 0.004), indicating that the association between age and career-related concerns differed by sex. Career-related concerns decreased with age among men but increased with age among women. This divergence likely reflects varying career trajectories. For men, professional advancement and stabilization of career position over time may alleviate early-career uncertainty. In contrast, for women, mid- to late career stages may coincide with cumulative structural barriers, including limited leadership opportunities and persistent childcare and caregiving expectations. In addition, women in our cohort were substantially less likely than men to have children and were significantly more likely to identify childbirth and childcare as barriers to career development. Although the reasons for these differences cannot be determined from this cross-sectional survey, these findings suggest that family-related factors may be closely associated with career development among women in neuro-oncology.

Job satisfaction with professional relationships was relatively high. This finding is consistent with previous literature showing that healthcare professionals often derive greater satisfaction from the collegial and relational aspects of work than from structural factors such as workload and compensation [8]. Long working hours were associated with lower satisfaction with income, working hours, and work-life balance, consistent with established evidence linking excessive workload to reduced well-being and burnout [9]. By contrast, holding a managerial/leadership position was associated with higher satisfaction, likely reflecting greater autonomy and perceived control. However, because managerial/leadership positions were largely occupied by older male respondents in our cohort, this association should be interpreted cautiously and may partly reflect differences in career stage rather than the independent effect of managerial/leadership position itself.

Female respondents were more likely to report perceived workplace discrimination, independent of working hours, caregiving experience, and managerial/leadership position. The persistence of this association after adjustment suggests that institutional culture and structural norms may contribute to gender disparities beyond measurable workload factors.

Although formal support systems for childbirth and caregiving were widely available, substantial perceptual gaps were evident between managerial and non-managerial staff. In Japan, national surveys have reported similar gaps between formal policy availability and practical utilization among women physicians [5]. Notably, 43.8% of respondents reported increased workload when colleagues took maternity, childcare, or caregiving leave, or used reduced working-hour arrangements. While most respondents expressed willingness to cooperate, a subset perceived the situation as unfair. Furthermore, long-hour workers were more likely to report both willingness to cooperate and perceptions of unfairness, suggesting a coexistence of professional commitment and emotional strain. These findings suggest that, without adequate staffing buffers and transparent workload redistribution, support policies may unintentionally shift the burden to colleagues. Sustainable implementation therefore requires organizational justice, leadership engagement, and structural workforce reform.

Overall, our findings underscore that gender equity in neuro-oncology cannot be addressed solely through individual-level interventions but instead requires coordinated institutional and systemic strategies. Structural measures addressing life course-related challenges—including transparent workload allocation, adequate staffing, and leadership development—may be particularly important for achieving sustainable workforce equity and professional fulfillment across genders.

## Limitations

This study had limitations. First, its cross-sectional design precludes causal inference. Second, the 35.9% response rate raises the possibility of response bias, as individuals with stronger opinions regarding gender or workplace issues may have been more likely to participate. Third, women comprised a relatively small proportion of respondents, which may have limited the statistical power of sex-based comparisons and reduced the precision of subgroup analyses. Fourth, the high proportion of participants in managerial/leadership positions (43.2%) may reflect preferential participation by senior members, potentially limiting the generalizability of the findings to early-career professionals. Fifth, outcomes were self-reported and may have been influenced by subjective perceptions. Sixth, this survey was conducted in 2021, before the implementation of physician work-style reforms in Japan. Perceptions regarding work environment, career development, and gender equity may have changed since the survey period. Finally, unmeasured institutional factors, such as hospital size, academic rank, and regional variation were not included in the analyses and may have confounded the observed associations. Despite these limitations, this nationwide survey provides valuable insight into the structural and cultural determinants of gender equity in neuro-oncology practice in Japan.

## Conclusions

In this nationwide survey of Japanese neuro-oncology professionals, significant sex disparities were observed in leadership representation, employment status, perceived discrimination, and career-related concerns. These disparities persisted independently of working hours and caregiving experience. Long working hours were associated with reduced job satisfaction, whereas managerial roles were associated with higher job satisfaction. Although formal support systems are widely available, perceptual gaps and workload redistribution challenges were evident. These findings highlight the need for coordinated institutional and system-level strategies to promote sustainable workforce development and gender equity in neuro-oncology.

## Supplementary Information

Below is the link to the electronic supplementary material.

Supplementary file1 (PPTX 81 kb)

Supplementary file2 (DOCX 25 kb)

## Data Availability

The data sets used and/or analyzed during this study are available from the corresponding author on reasonable request.
