# Supplementary material for: Working conditions, career barriers and perceived equity among neuro-oncology professionals: insights from the Japan society for neuro-oncology (JSNO) gender equality and diversity committee survey
Source: Int J Clin Oncol. 2026 Aug 18;31(11):2627–34. doi: 10.1007/s10147-026-03168-y (PMC13710562; doi:10.1007/s10147-026-03168-y)
Supplement: Supplementary file 1 [file 10147_2026_3168_MOESM2_ESM.docx]

Supplementary Appendix

Questionnaire for the JSNO Gender Equality and Diversity Committee Survey

1. Please indicate your profession. **(Select all that apply.)**
・Neurosurgeon
・Pediatrician
・Radiologist
・Pathologist

・Other physician
・Researcher (Physician)
・Researcher (Non-physician)
・Nurse
・Physical Therapist / Occupational Therapist / Speech-Language Therapist
・Social Worker
・Psychologist
・Other: __________

2. Sex **(Select one.)**
・Male
・Female

3. Please enter your age as of August 31, 2021: __________

4. Years since graduation: __________ years

5. Do you hold board certification in your specialty? **(Select one.)**
・Yes
・No
・Not a physician

6. Are you currently employed? **(Select one.)**
・Yes
・No

For respondents who are currently employed (7-17):
7. What is your current employment status? **(Select one.)**
・Full-time
・Part-time (including temporary, contract, or other part-time positions)
・Full-time employee with reduced working hours
・Fixed-term contract employee
・Temporary agency employee

8. What is your current position? **(Select one.)**
・Professor
・Associate Professor
・Lecturer
・Assistant Professor
・Department Chair / Department Head
・Chief Physician
・Staff Physician
・Resident / Trainee
・Graduate Student
・Other: __________
 
9. How many hours do you work per week (actual working hours)?
__________ hours

10. How many night shifts do you work per month?
__________ shifts

11. Is there a daycare center or childcare facility at your workplace? **(Select one.)**
・Yes
・No
・Not sure

12–17. Job satisfaction across six domains.
Please indicate your level of satisfaction with each of the following domains of your current job. (Select one response for each domain.)

Response options:

・Very satisfied

・Satisfied

・Neutral

・Dissatisfied

・Very dissatisfied

Domains
12. Income
13. Position/status

14. Working hours
15. Use of skills and abilities
16. Work-life balance
17. Relationships with supervisors and colleagues

18. Please indicate all family members currently living with you. (Select all that apply.)
・Spouse/Partner
・Parent(s) (including parent(s)-in-law)
・Other household member(s) (including sibling(s))
・Child(ren)
・I live alone (including living apart from my family due to work)

19. Who is primarily responsible for household chores in your household?

(Select all that apply.)
・Myself
・Spouse/Partner
・Parent(s) (including parent(s)-in-law)
・Other household member(s) (including sibling(s))
・Child(ren)
・Other: __________

20. What is your marital status? **(Select one.)**
・Never married
・Married
・Divorced/Widowed

21. For respondents who respond being married or divorced/widowed:

Did your employment status change after marriage? **(Select one.)**
・Yes
・No
 
22. Do you have concerns regarding your career development? **(Select one.)**

・Yes
・No
・Not sure

23.　What do you consider to be barriers to your career development? (Select all that apply.)
・Physical condition or stamina
・Personal abilities or skills
・Poor working conditions
・Lack of access to professional education at the workplace

・Workplace interpersonal relationships
・Difficulty finding suitable employment
・Marriage
・Childbirth and childcare
・Children's education
・Workplace gender discrimination
・Lack of family support
・Spouse's job transfer or overseas assignment

・Financial issues
・Other: __________

24. What types of support would you like to receive to help your career development?

(Select all that apply.)
・Clearer working conditions (e.g., working hours and salary)
・Clearer employment status and benefits (e.g., health insurance and pension)
・Adoption of a team-based care system instead of an individual primary physician system
・Greater flexibility in postgraduate training programs
・Better opportunities for research and overseas study
・On-site childcare facilities
・Better childcare services for sick children
・Job-sharing opportunities
・Flexible working hours (flextime)
・Redistribution of responsibilities among multidisciplinary healthcare professionals
・Availability of backup staff for emergencies
・Greater awareness and support in the workplace
・Greater understanding and support from family
・Employment information and professional networking opportunities
・Re-entry training programs after a leave of absence
・Re-employment support after a leave of absence
・Other: __________

25. Have you experienced discrimination in your workplace? (Select one.)
・Very strongly

・Often

・Neutral

・Rarely

・Not at all

26. Have you experienced discrimination regarding promotion opportunities? **(Select one.)** 
・Very strongly

・Often

・Neutral

・Rarely

・Not at all

27. Have you ever taken a leave of absence from work?
**(Select one.)** 
・Yes
・No

For respondents who have taken a leave of absence (28-30):

28. How many years after graduation did you first take a leave of absence?
 __________ years

29. How long was your leave of absence? (Please specify the duration in years and months.)

30. What were the main reasons for taking a leave of absence? (Select all that apply.)
・Loss of motivation
・Uncertainty about my career or future
・My own illness or health condition
・Wishes or expectations of family/relatives
・Interpersonal relationships
・Heavy physical workload
・High psychological stress
・Marriage
・Pregnancy
・Childbirth
・Childcare
・Financial reasons
・Caregiving for a family member
・Working conditions (including overnight/on-call duties)
・Other: _________

31. Do you have any children? **(Select one.)** 
・Yes
・No

For respondents who reported having children (32-41):
32. How many dependent children do you currently have?
 
33. Please indicate the ages of all your dependent children.

34. Who are the primary caregivers for your child(ren)? (Select all that apply.)
・Myself
・Spouse/Partner
・Parent(s) (including parent(s)-in-law)
・Other household member(s) (including sibling(s))
・Child(ren)
・Other: __________

35. Have you ever taken maternity or paternity leave? **(Select one.)** 
・Yes
・No
・I wanted to, but I was unable to

36. Have you ever taken parental leave? **(Select one.)** 
・Yes
・No
・I wanted to, but I was unable to

37. Did your employment status change after the birth of your child(ren)? **(Select one.)** 
・Yes
・No

38. During your regular working hours, who or what primarily cares for your child(ren)? (Select all that apply.)
・After-school care program
・Licensed daycare center
・Unlicensed daycare center
・Workplace childcare facility
・Grandparent(s)
・Spouse/Partner
・Relative(s) or sibling(s)
・Babysitter
・Friend(s), acquaintance(s), or neighbor(s)
・Family Support Center (community childcare support service)
・Extracurricular activities
・Not an infant or school-aged child
・Other: __________

39. Problems encountered while working
Have the following issues been a problem for you while working?
For each of the following items, please indicate whether it has been a problem by selecting one of the following responses: Yes, No, or Not applicable.

・Admission to a licensed daycare center
・Arranging care when a child becomes ill
・Child's infectious illnesses
・Child's chronic illness
・Child's extracurricular activities
・Child's education
・Child's psychological or emotional problems
 ・Daycare center events
・School events
・Class or school closures
・Long school vacation periods

40. Please provide details about the problems you encountered.

41. How did you deal with these problems? (Select all that apply.)
・Relied on my parent(s) or parent(s)-in-law
・Used an unlicensed daycare center
・Used a childcare service for sick children
・Hired a babysitter
・Used a Family Support Center service
・Took time off from work myself
・My spouse/partner took time off from work
・Left my job
・My spouse/partner left their job
・Other: __________

42. Caregiving experience
Have you ever provided care for a family member requiring long-term care? **(Select one.)** 
・Yes
・No

For respondents with caregiving experience(43-44):

43. Who are the primary caregivers? (Select all that apply.)
・Myself
・Spouse/Partner
・Parent(s) (including parent(s)-in-law)
・Other household member(s) (including sibling(s))
・Child(ren)
・Other: __________

44. Have you ever taken caregiving leave? **(Select one.)** 
・Yes
・No

45. Do you think reduced working hours are necessary for colleagues (regardless of gender) with pregnancy-, childcare-, or caregiving-related responsibilities? **(Select one.)**

・Necessary

・Not necessary

・Neutral

46. Have you experienced a substantial increase in your workload due to colleagues (regardless of gender) taking maternity leave, childcare leave, caregiving leave, or using reduced working-hour arrangements? **(Select one.)**

・Yes
・No
・Unsure

47. If you answered "Yes" to the previous question, how did you feel about this situation? (Select all that apply.)
・I felt willing to cooperate.
・I felt the situation was unfair.
・I did not feel anything in particular.
・Other (please specify):

48. In your opinion, what measures are needed to ensure that parental leave, caregiving leave, and family support programs can be used effectively while maintaining a fair distribution of workload among staff? Please describe your thoughts.

49. Do you think your workplace is supportive of employees who are raising children or providing caregiving? **(Select one.)** 
・Yes
・No
・Not sure

50. Are the following policies or support systems for pregnancy, childbirth, childcare, and caregiving available at your workplace?
For each item, please select one of the following responses: Yes, No, or Not sure.

Item
・Maternity leave
・Commuting accommodations and health checkups during pregnancy
・Exemption from night duty during pregnancy
・Substitute staff during maternity leave
・Salary payment during maternity leave
・Employment protection during maternity leave

・Childcare leave system
・Substitute staff during childcare leave

・Employment protection during childcare leave
・On-site or nearby daycare/childcare facilities
・Night-time childcare services
・Sick-child care system
・On-site or nearby after-school childcare facilities
・Caregiving leave system
・Substitute staff during caregiving leave
・Employment protection during caregiving leave

・Gender equality office, support desk, or mentoring system

・Reduced-hours full-time employment
・Reduced-time regular employment

・Exemption from radiation-related duties

51. What policies, systems, or support measures do you think are necessary to help balance work with childcare or caregiving responsibilities? (Select all that apply.)
・Increase in the number of physicians

・Increase in non-physician staff

・Review of the primary physician system (e.g., multiple-attending-physician model)

・Two-shift work system
・Assignment of substitute physicians
・Employment of substitute staff
・Exemption from night/on-call duty
・Exemption from overtime work
・Expansion of reduced working-hour employment
・Introduction of flexible working systems
・Transfer to more accommodating departments
・Development or expansion of childcare centers or nurseries
・On-site childcare facilities
・Sick-child care services
・Dispatch of childcare facilities or babysitters
・Financial support for childcare facility use
・Financial support for babysitter use
・Expansion of after-school childcare programs
・Promotion of men’s participation in housework/childcare/caregiving
・Support for returning to work after maternity/childcare/caregiving leave
・Expansion of government subsidy programs for workplaces
・Expansion of long-term care facilities
・Support for returning to work after caregiving leave
・Financial support for domestic helper services
・Home-based or remote training systems
・Increase in long-term care reimbursement or social security expenditure
・Increase in medical service fees or healthcare expenditure
・Other

52. Whom would you like to consult about your future career? (Select all that apply.)
・A senior physician in my specialty / a senior colleague in my profession
・A physician or professional colleague of my generation
・My supervisor
・Friends outside the medical profession or friends in other professions
・The Gender Equality Committee of the Japan Society for Neuro-Oncology
・I would not like to consult anyone
・My family　
・Other (please specify): __________

53. Do you collaborate with other healthcare professionals in your work? **(Select one.)** 
・Yes
・No
・Not sure

54. In your opinion, what is needed to promote multidisciplinary collaboration in basic and clinical neuro-oncology? Please describe your thoughts.

55. Which of the following activities would you like the Gender Equality Committee of the Japan Society for Neuro-Oncology to undertake? (Select all that apply.)
・Policy recommendations to promote gender equality in basic and clinical neuro-oncology research
・Research awards for women physicians
・Research funding for women physicians serving as principal investigators or leading multidisciplinary collaborative research
・Sharing best practices for promoting gender equality and multidisciplinary collaboration in basic and clinical neuro-oncology research
・Establishment of a mentoring program to promote gender equality in basic and clinical neuro-oncology research
・Other (please specify): __________

56. If you have any expectations or suggestions for the Gender Equality Committee of the Japan Society for Neuro-Oncology, please describe them below.
